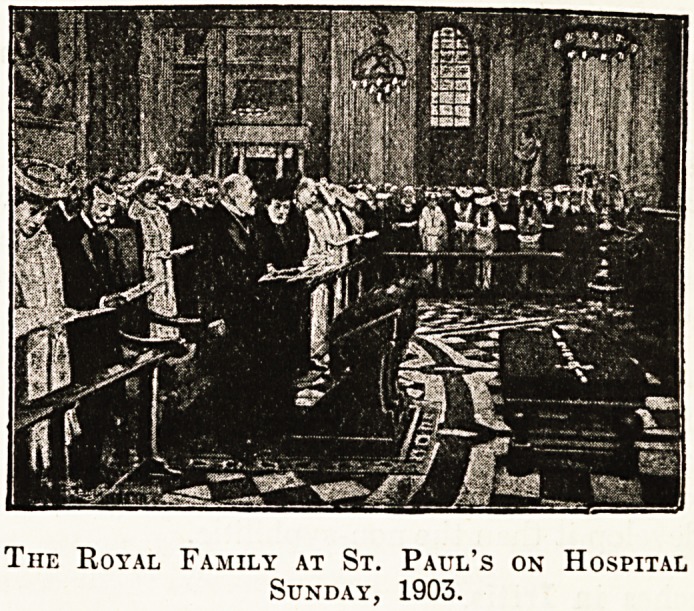# The King and Queen Privately Attend the Morning Service

**Published:** 1912-06-15

**Authors:** 


### HOSPITAL SUNDAY AT ST. PAUL'S.

### The King and Queen Privately Attend the Morning Service.

HOSPITAL Sunday in 1912 will stand out in the annals of the Sunday Fund movement from the fact that the King and Queen attended the morning service in St. Paul's Cathedral.

Their visit was a private and personal one. It was robbed of all ceremony, the better, no doubt, to emphasise the great example which their Majesties set to every congregation in London of attending on this Sunday in the year. It would, indeed, be hard to exaggerate the true significance of this private attendance at London's chief church. Hospital Sunday could have had no finer and certainly no more practical proof of Royal approval. It is a visit that will never be forgotten as succeeding Hospital Sundays come round.

We are, therefore, glad to be privileged to publish an illustration of their Majesties' entry into the Cathedral, for the simplicity which it reveals may be mirrored as well as described. But the illustration itself gains added significance by comparison with that which we were enabled to publish on a previous occasion, when in 1903 King Edward and the two illustrations provides a unique record of the permanent interest in Hospital Sunday which

THE ROYAL FAMILY AT ST. PAUL'S ON HOSPITAL SUNDAY, 1903.

is taken by our Royal Family. That interest has become a tradition, and every hospital-worker in all

[*Photograph by* *Central News*].

THE KING AND QUEEN RECEIVED BY DEAN INGE AT ST. PAUL'S.

Queen Alexandra attended St. Paul's in State on the afternoon of Hospital Sunday. Their present Majesties were present as Prince and Princess of Wales on that occasion, and the juxtaposition of the varied fields of institutional life will appreciate the record that is so tersely but so forcibly expressed in these illustrations set side by side after an interval of almost ten years.

## Figures and Tables

**Figure f1:**
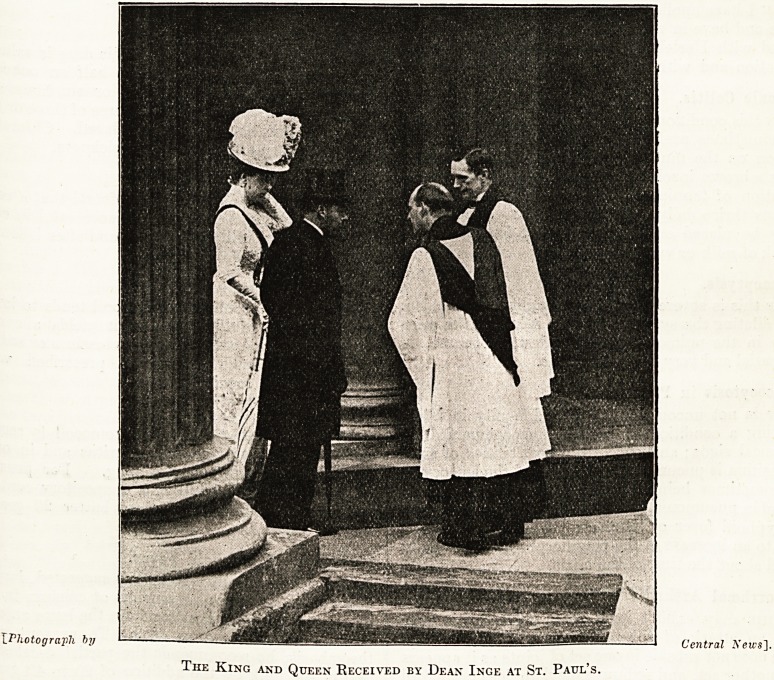


**Figure f2:**